# Agreement between diagnoses reached by clinical examination and available reference standards: a prospective study of 216 patients with lumbopelvic pain

**DOI:** 10.1186/1471-2474-6-28

**Published:** 2005-06-09

**Authors:** Mark Laslett, Barry McDonald, Hans Tropp, Charles N Aprill, Birgitta Öberg

**Affiliations:** 1 Dept for Health and Society: Physiotherapy, Linköping University, Linköping, Sweden; 2 Institute of Information and Mathematical Sciences, Massey University, Albany, New Zealand; 3 Dept for Health and Society, Linköping University, SE-58183 Linköping, Sweden; 4 Louisiana State University Health Science Center, 2718 Cadiz St, New Orleans, LA 70115, USA; 5 SwedenDept for Health and Society, Linköping University, SE-58183 Linköping, Sweden

## Abstract

**Background:**

The tissue origin of low back pain (LBP) or referred lower extremity symptoms (LES) may be identified in about 70% of cases using advanced imaging, discography and facet or sacroiliac joint blocks. These techniques are invasive and availability varies. A clinical examination is non-invasive and widely available but its validity is questioned. Diagnostic studies usually examine single tests in relation to single reference standards, yet in clinical practice, clinicians use multiple tests and select from a range of possible diagnoses. There is a need for studies that evaluate the diagnostic performance of clinical diagnoses against available reference standards.

**Methods:**

We compared blinded clinical diagnoses with diagnoses based on available reference standards for known causes of LBP or LES such as discography, facet, sacroiliac or hip joint blocks, epidurals injections, advanced imaging studies or any combination of these tests. A prospective, blinded validity design was employed. Physiotherapists examined consecutive patients with chronic lumbopelvic pain and/or referred LES scheduled to receive the reference standard examinations. When diagnoses were in complete agreement regardless of complexity, "exact" agreement was recorded. When the clinical diagnosis was included within the reference standard diagnoses, "clinical agreement" was recorded. The proportional chance criterion (PCC) statistic was used to estimate agreement on multiple diagnostic possibilities because it accounts for the prevalence of individual categories in the sample. The kappa statistic was used to estimate agreement on six pathoanatomic diagnoses.

**Results:**

In a sample of chronic LBP patients (n = 216) with high levels of disability and distress, 67% received a patho-anatomic diagnosis based on available reference standards, and 10% had more than one tissue origin of pain identified. For 27 diagnostic categories and combinations, chance clinical agreement (PCC) was estimated at 13%. "Exact" agreement between clinical and reference standard diagnoses was 32% and "clinical agreement" 51%. For six pathoanatomic categories (disc, facet joint, sacroiliac joint, hip joint, nerve root and spinal stenosis), PCC was 33% with actual agreement 56%. There was no overlap of 95% confidence intervals on any comparison. Diagnostic agreement on the six most common patho-anatomic categories produced a kappa of 0.31.

**Conclusion:**

Clinical diagnoses agree with reference standards diagnoses more often than chance. Using available reference standards, most patients can have a tissue source of pain identified.

## Background

Different pathoanatomic conditions and mechanisms in the lumbar spine and pelvis region may produce low back pain (LBP), pelvic or lower extremity symptoms. The most frequent tissue sources of lumbopelvic and referred pain are the intervertebral discs, zygapophysial (facet) and sacroiliac joints [1]. Nerve root irritation, spinal stenosis, the hip joint, fractures, neoplasms and disorders of the vascular system or viscera are potential but less common sources of lumbopelvic or lower extremity pain. Psychosocial distress increases the complexity of diagnosis, confounds therapeutic endeavours and is a major factor determining disability [2,3]. During the last 20 years, advances in technology and clinical science have resulted in techniques that have improved our ability to identify the tissue origin of LBP and referred lower extremity symptoms. While controversy surrounds techniques such as provocation discography for diagnosis of discogenic pain [4-6], the value of diagnostic anaesthetic blocks to the lumbar zygapophysial joints (ZJ) and sacroiliac joints (SIJ) is more secure. A body of evidence supports the use of these diagnostic procedures in specific clinical circumstances using established methodological guidelines [7-13]. While it is commonly stated that no pathoanatomic explanation for symptoms is possible for about 80% of cases [2,14], some now argue that the use of recently improved diagnostic and clinical reasoning techniques makes diagnosis of the tissue origin of pain possible in 46–75% of cases [7]. Because these procedures are invasive, they cannot be justified in acute or subacute cases, since prognosis in these cases is good [7]. However there is value in such a diagnosis for chronic LBP. Between 5 and 10% of patients initially visiting a primary care physician for LBP will ultimately develop chronic LBP [15]. These patients have a high level of dissatisfaction with primary care management [16] yet continue to desire a diagnosis and explanation for persistent pain and disability [17]. Persistent discogenic pain may be treated with spinal fusion or intradiscal electrothermal annuloplasty (IDET), ZJ pain with intra-articular injections or medial branch neurotomy, and SIJ pain with intra-articular injections or surgical fusion. These procedures are invasive, and are not universally successful in returning the patient to pain-free full function. While psychosocial distress undoubtedly plays an important role in these less than ideal outcomes, poor pre-procedure selection and diagnosis, is a major contributing factor to failure [7].

The clinical examination (history and physical examination with or without imaging) is the basis upon which management rests. The diagnostic value of this examination is debatable for all but a minority of cases. A few symptomatic patho-anatomic entities such as disc herniations causing nerve root compression, symptomatic spinal stenosis, radiologically demonstrated fractures and neoplasms may be identified using the clinical examination in combination with advanced imaging studies. The current project was conceived to compare diagnoses derived from a detailed clinical examination by a physiotherapist, with expert diagnoses obtained using available reference standards for diagnosis of discogenic, facetogenic, SIJ, hip joint, nerve root pain and symptomatic spinal stenosis.

## Methods

**Figure 1:**
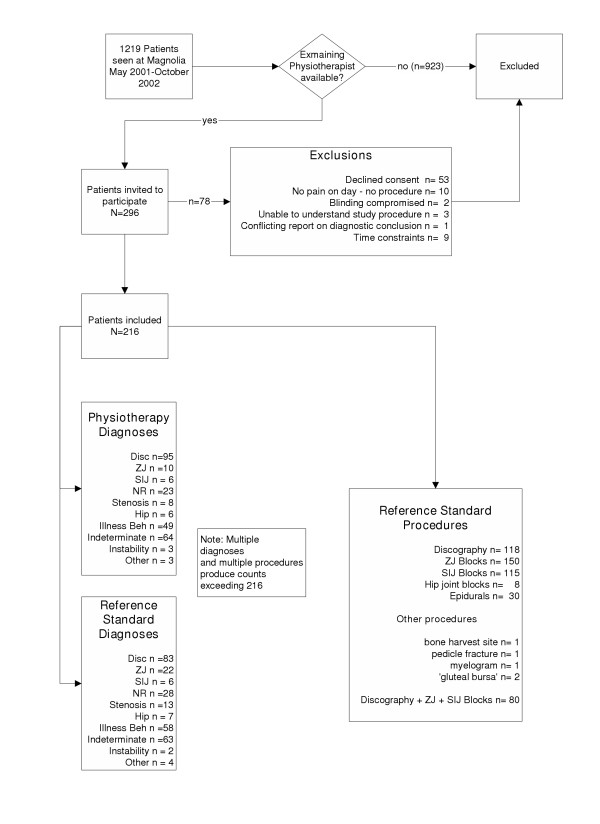
Summary of patient recruitment, diagnoses by examiners and reference standard procedures used.

A prospective, blinded, reference standard-related design was utilized. The Louisiana Institutional Review Board approved the study and all included patients signed an informed consent form. A physiotherapist with 30 years experience as a manipulative therapist attended a specialist spinal diagnostic clinic in Louisiana, for blocks of 4–8 weeks between May 2001 and October 2002 and examined consecutive chronic LBP patients during these periods. Clinical examinations required between 30 and 60 minutes and were carried out immediately before the reference standard diagnostic tests. A radiologist with 20 years experience in fluoroscopically guided diagnostic injections and interpretation of advanced imaging techniques attempted to identify the tissue origin of chronic LBP, based on imaging and responses to diagnostic injections. These diagnoses were the reference standards against which diagnoses arrived at by the clinical (physiotherapy) examination were contrasted. Another therapist with 17 years clinical experience carried out examinations of 13 patients. Figure 1 presents a flow diagram describing patient recruitment, summary of examiner's diagnoses and reference standards employed.

The great majority of possible causes of LBP are uncommon, even rare. Appendix 1 presents those painful clinical entities believed to be most common and the procedures used as diagnostic reference standards [see additional file 4].

In addition, three other categories were available to the examining clinicians – "illness behaviour" where the patient's behaviour and responses to questioning and examination suggested psychosocial distress of some kind, "others" (for the more uncommon causes of pain) and an "indeterminate" category where no conclusion could be reached.

At presentation, clinic staff collected medical history, demographic and questionnaire data. If informed consent was obtained, the physiotherapist examined the patient and the patient received scheduled diagnostic injection procedures in sequence.

### Blinding

The physiotherapy examinations were conducted blind to the results of disability and self report questionnaires, the results of previous imaging studies and diagnostic injections. The physician was blinded to the results of the physiotherapy examination and diagnostic conclusions.

### Diagnostic classification

The clinical reasoning by which the physiotherapist reached a diagnosis has been presented elsewhere in detail [18,19]. Very briefly, discogenic pain was concluded when centralisation, peripheralisation [20-22] or directional preference [23] were reported by the patient during an examination with repeated standardised end range test movements [24], or if the dominant or primary pain was located in the exact midline of the lumbar spine. ZJ pain was recorded if the clinical criteria specified by Revel et al (1998) [25] were satisfied in the absence of centralisation. SIJ pain was recorded if three or more stress SIJ tests [26] provoked familiar pain in the absence of centralization [27,28]. Nerve root pain was recorded when referred pain was provoked with nerve tension tests. Symptomatic spinal stenosis was recorded when the patient reported a clear pattern of intermittent claudication which was relieved by sitting or a flexed spinal posture [29]. Hip joint pain was recorded if passive movements of the hip provoked familiar pain more readily than SIJ provocation or lumbar tests [30,31]. Diagnosis of instability presented a problem since no reference standard exists [7,8,32,33]. The radiologist's diagnosis was based on the observation of paradoxical motion on flexion / extension radiographs [34,35] and the physiotherapy clinical diagnosis was based on clinical criteria gleaned from post graduate course material and published opinion [32,36,37].

Some data on the diagnostic accuracy of the clinical tests used in the physiotherapy clinical examination was available prior to commencement of the study. A summary for key tests is presented in Appendix 2 [see additional file 5].

The physiotherapist and physician recorded their diagnoses on standardised forms. In January 2003 a meeting of researchers and experienced clinicians was convened at the Auckland University of Technology to recommend the method by which the diagnosis data were to be entered into an electronic database for analysis.

### Data analysis

Data were entered and stored in an electronic database (Minitab version 14.12 ^© ^2001). Two variables were constructed to record agreement between physiotherapy and reference standard diagnoses. "Exact agreement" was recorded when the two diagnoses were exactly the same in all respects, including multiple diagnoses. "Clinical agreement" was recorded when the physical therapy diagnosis was included within the reference standard diagnosis (e.g. if the physiotherapy diagnosis was ZJ pain and the reference standard diagnosis was ZJ pain and symptomatic spinal stenosis). All cases of 'exact' agreement are included within 'clinical' agreement. Confidence intervals (CI) for proportions were calculated using recommended methods [38]. Agreement between physiotherapy diagnoses and reference standard diagnoses was estimated using the kappa statistic, which accounts for chance agreements. Kappa values of zero reflect chance agreement, values less than 0.0 reflect agreement worse than chance with -1.0 representing perfect disagreement. Values greater than 0.0 reflect agreement better than chance agreement with +1.0 representing perfect agreement [39]. The kappa statistic for multiple categories was calculated using Confidence Interval Analysis Software ^© ^T Bryant 2000 [40]. Chance agreement is strongly influenced by the prevalence of the diagnostic category. The more prevalent the disorder, the more likely the physiotherapist would correctly match the reference standard diagnosis on the basis of chance. To estimate agreement while accounting for the influence of disease prevalence and the number of possible diagnoses, the rule-of-thumb "proportional chance criterion" (PCC) was used. The PCC is commonly used in discriminant analysis to judge whether a classification method is better than guessing. The PCC is the expected proportion of correct classifications [41], and equals the sum of squared prevalences. Standard errors (SE) for PCC and agreement were calculated as (sqr(p(1-p)/n)) and 95% confidence intervals calculated as: proportion ± (1.96 * SE) [42].

To simply the evaluation of agreements on patho-anatomic diagnoses, both physiotherapy and reference diagnoses were condensed to six categories: disk, facet (z joint), sacroiliac joint, nerve root, hip joint and spinal stenosis. Where more than one diagnosis was provided, the case was included in each diagnostic category. For example: If the physiotherapist diagnosis was: disk and nerve root, the counts for both disk pain and nerve root pain were incremented by one. If the reference standard diagnosis was disc, nerve root and spinal stenosis, each of these diagnostic categories were incremented by one. Consequently there were many more diagnoses than patients.

## Results

During the study period, 296 patients were invited to participate in the project and 78 were excluded. Reasons for exclusion were: 53 declined to participate, ten had no injection or other procedure carried out because of insufficient pain on the day of examination, nine were excluded because of time constraints, three patients were deemed incompetent to understand study procedure, blinding was compromised in two cases, and contradictory diagnostic reporting of diagnostic injection results occurred in one case. Table 1 presents demographic, historical and profile data for included patients (n = 216) [see additional file 1]. Figure 1 presents patient recruitment patterns in the project.

Table 2 presents a cross-tabulation of physiotherapist diagnoses with diagnoses reached by the interventional radiologist [see additional file 2]. The radiologist came to a single diagnostic conclusion in 144 cases (66%) and more than one in 72 (34%) cases, with two cases having three diagnoses. The physiotherapist reached a single diagnostic conclusion in 163 (76%) of cases and two conclusions in 53 cases. Based on reference standard / expert opinion diagnoses, the chance of the physiotherapist correctly guessing the diagnosis (PCC) was 13% with 95% confidence intervals of 9% and 18% (standard error = 0.023). Exact agreement (standard error, 95% CI) achieved was 32% (0.032, 26%, 38%) and clinical agreement 51% (0.034, 45%, 58%).

Many of the diagnostic categories contained only one case, so diagnoses were grouped under nine general labels: disc, Z-joint, sacroiliac joint, nerve root, hip joint, spinal stenosis, "other", "illness behaviour" and "Indeterminate". A total of 368 diagnoses were provided by physiotherapy and reference standard clinicians through multiple diagnoses. "Illness behaviour" was the sole diagnostic conclusion or included in 79 (21.5%) of 368 conclusions by the reference standard clinician. The physiotherapy examiner used this description for 76 cases. "Indeterminate" was included in 84 cases by the reference standard clinician and in 91 cases by the physiotherapist. No reference standard for illness behaviour was established *a priori *in this study and "indeterminate" is the absence of a diagnosis. The primary objective was to evaluate agreement on patho-anatomic diagnoses. To evaluate agreement on diagnosis for the six main patho-anatomic diagnoses: discogenic pain, Z-joint pain, sacroiliac joint pain, nerve root (radicular) pain, hip joint pain and spinal stenosis, Table 3 was truncated (represented by the dashed lines), by removing the columns and rows for the other categories [see additional file 3]. The category "Other" was removed from this analysis as it contained some uncommon pathologies: a rhabdomyosarcoma affecting the psoas and hip, a symptomatic spondylolysis, bone graft donor site pain, nerve root irritation from surgical hardware, and vascular claudication secondary to peripheral artery disease. After removal of the columns and rows representing non-patho-anatomic diagnoses and 'Others", 137 patho-anatomic physiotherapy and reference standard diagnoses. The chance that the physiotherapist would correctly guess the diagnostic category (PCC, standard error, 95% CI) was 33%, 0.039, 26%, 41%). Agreement achieved (standard error, 95%CI) was 57% (0.041, 48%, 64%). Kappa statistic (standard error, 95% CI) for this table is 0.31 (0.067, 0.18, 0.44).

## Discussion

To our knowledge, this is the first study to estimate agreement between diagnoses based on blinded clinical (physiotherapy) examinations, and a range of diagnoses using available reference standards and other classification categories, in patients presenting with pain presumably of lumbopelvic origin. Overall agreement was better than could be expected on the basis of chance and ranged from 32% to 57% depending on agreement criteria and complexity of the diagnostic categorization analyzed. The proportion of patients indeterminate to reference standard diagnostic methods was 23%. However, the categories "illness behaviour", "indeterminate" and "instability" are non-pathoanatomic i.e. 'non-specific' in origin. There were 73 cases (34%) that the reference standard clinician classified using only these descriptions, and may be considered patho-anatomically 'non-specific'. This figure corresponds with claims that 46–75% of LBP patients have an identifiable tissue origin of pain using a reductionist approach to diagnosis [7].

The kappa statistic measures strength of agreement between examiners discounting chance agreement. The achieved level of 0.31 is considered 'fair agreement' [43]. However the context of this project is important. The patients in this sample were referred for invasive diagnostic testing and were typically chronic with high levels of distress and disability. Most patients had failed multiple attempts at treatment, and many had seen a number of general and specialist clinicians without a satisfactory diagnosis being provided. This was anticipated prior to commencement of the data collection phase of the project, and it was accepted that psychosocial distress would impact on the ability of physiotherapy and reference standard clinicians to make a tissue specific diagnosis. Some 87 patients (40%) had "illness behaviour" or "indeterminate" as the sole description, or as a part of the diagnostic classification provided by the reference standard clinician. The single greatest cause of "indeterminacy" in reference standard classifications was "illness behaviour". A similar pattern emerged for the physiotherapy clinical classifications (Tables 2 and 3). Indeterminacy and illness behaviour combined, accounted for as many patients as the largest pathoanatomic diagnostic group (disc). There was no reference standard for "illness behaviour" established prospectively, so any agreement between the physiotherapist and physician is merely interesting. Cases that were diagnostically indeterminate without apparent "illness behaviours" were also a large group, constituting 28/216 and 32/216 for the interventional radiologist and physiotherapist respectively. This is a result that might be expected in a tertiary referral environment, but was due in part because a proportion of patients could not receive the full battery of interventional and clinical tests needed to arrive at a patho-anatomic diagnosis. Restrictive terms of referral and low patient tolerance to discomfort were the primary reasons for failure of patients to receive all appropriate tests. Time constraints limited the physiotherapy examination procedure in less than 5% of cases included in the analysis. Cost containment was not a factor limiting reference standard or physiotherapy examination. The physiotherapy examination was provided free and any additional interventional procedures over and above those recommended on referral were also provided free.

The data provides information about agreement on diagnosis between non-invasive and inexpensive clinical methods of diagnosis carried out by a physiotherapist and a radiologist using invasive and expensive diagnostic technology, over and above chance. Across the whole spectrum of possible reasons for patients attending a tertiary referral diagnostic clinic estimated exact agreement is 19% (32% versus 13%) over and above chance. In practical terms, exact agreement is not required or expected when examining complex patients. 'Clinical agreement' is a more appropriate measure in that it is a measure of the degree to which the physiotherapist can reach accord with at least one part of the diagnosis. Clinical agreement is also estimated to be 19% better than chance agreement. For patho-anatomic categories, agreement over chance is 24% (57% versus 33%). The greater agreement on the six patho-anatomic categories may be a result of clearer diagnostic criteria for categorization by both clinicians. Some discordance between the physiotherapy and reference standard diagnoses resulted from one examiner being able to reach a diagnosis where the other was unable to carry out the examination(s) needed. Restrictions inherent in some referrals, meant that with some cases, the physiotherapist was able to reach a diagnosis, but the radiologist could not use the appropriate procedure necessary to provide a diagnosis for comparison. Conversely, some patients could not tolerate some parts of the physiotherapy clinical examination, whereas a clear diagnostic result was possible using interventional diagnostic procedures. These cases of unilateral indeterminacy resulted in disagreements, whereas agreement may have been possible if both clinicians could have fully examined the patients.

It was anticipated was that discogenic pain cases would be more numerous than other tissue sources of pain with 85 (39%) receiving this diagnosis and 59 (27%) having this as the sole diagnosis. This proportion is in concord with previous results [44]. Fewer ZJ and SIJ cases were identified than expected with significant consequences. Estimates of agreement between the physiotherapy and reference standard diagnoses for the less frequent diagnoses, especially SIJ pain, were compromised. The numbers for these less frequently occurring conditions are too low to enable conclusions to be drawn from the results. The identification of hip joint pain among patients referred as LBP sufferers can be expected [45,46], but it was not anticipated that they would be more prevalent than SIJ cases (Table 2).

In this sample 22 cases (10%) received two patho-anatomic diagnoses (by available reference standards), and two had three identified pain generators (Table 2). To our knowledge only one other study has reported multiple sources of nociceptive input to low back pain with about 3% having co-existent disc and ZJ pain [47]. In the current study, only two patients had discogenic and facetogenic pain (1%).

An issue for this study concerns generalizability. The patients were chronic and distressed and nearly 30% of included patients had a history of lumbar spinal surgery with persistent or recurrent pain. Physiotherapists do see patients in all stages and degrees of LBP and our results are arguably generalizable to more chronic patient populations. We did not exclude patients with a history of spinal surgery, so this study may be more representative of tertiary care patient samples than other back pain studies. Generalizability to acute and subacute LBP populations is not appropriate, but at the conceptual level at least, it may be argued that acute and subacute patients would be less complex than patients in this study. Consequently our results may represent the lower bound of potential agreement between physiotherapy diagnostic conclusions and reference standard diagnoses.

Another issue involving generalizability concerns the examining physiotherapists. Are the diagnostic techniques and procedures used, representative of a special interest "craft" group and not within the normal domain of practicing clinicians? Physiotherapists have a wide range of special interests generally and there are many schools of thought within musculoskeletal practice. In the last 15 years, an 'extended practice' role for physiotherapists in orthopaedic musculoskeletal practice has emerged [48] requiring more advanced training than is required to conduct the examination used in this study [49-51]. In this study the examination techniques of concern are the McKenzie repeated movement's examination, the provocation SIJ tests and the tests used by Revel et al (1998). The McKenzie system has been in the public domain since 1981 [20] and is the most widely used system among therapists in North America for examining and treating LBP patients [52]. It has been formally studied for inter-examiner reliability [53] with satisfactory results among trained clinicians [54,55] and for validity [22,23,56]. The provocation SIJ tests have been in the public domain for many decades [30] and the principal author has examined them for reliability [26] and validity [28] with satisfactory results. The criteria used to identify patients suitable for screening ZJ blocks ("Revel's criteria") are simple and well documented [25], although recently these authors' results have not been replicated [57,58]. The feasibility, reliability and construct validity of the clinical reasoning and classification system has been evaluated [19,59]. It is our contention that the system may be generalized to a proportion of clinicians, or can be learned readily enough at post-graduate level.

The use of discography, ZJ and SIJ blocks as reference standards may be criticized on the basis that false positives may compromise their status as "gold standards" [60,61]. It is recognized that perfect gold standards do not exist for the diagnosis of discogenic, facetogenic pain or pain arising from the SIJ [62]. However, modern methodology accounts for these potential errors and we believe that no alternative satisfactory standards exist [7].

### Implications for clinical practice and future research

A comprehensive history and physical examination similar to that utilized in the current project has some potential to predict the diagnostic conclusions reached by an experienced physician using sophisticated and invasive technologies. Most of the agreement calculated was made up from identifying pain originating from the lumbar discs and the hip joints. Previous research has shown that pain arising from the ZJ joints cannot be characterized by clinical examination variables [57,58,63], and the results of this study support this conclusion. Although the current data does not permit a direct test of the validity of the clinical examination in relation to pain known to arise from SIJs, recent previous work has indicated that the clinical reasoning process and the examination techniques have some validity and clinical utility [28].

The diagnosis of instability is fraught with conceptual and terminological difficulties without widely accepted clinical diagnostic criteria [8,32,64]. In this study some historical cues and clinical findings suggestive of this condition [36] were documented and some results will be reported (Laslett, Oberg et al Submitted October 2004)

Nerve root pain was the second most common tissue-based diagnosis made by both physiotherapist and radiologist after discogenic pain. Although conceptually and clinically distinct, radiculopathy (clinical evidence of sensory and/or motor deficit) and radicular pain (lancinating pain in a myotomal distribution secondary to axonal stimulation rather than nociceptive stimulation) were combined in the diagnosis of "nerve root pain". It was differentiated from somatic referred pain believed to result from central nervous system convergence [8]. In this study, pain arising from the lumbar nerve roots was not consistently diagnosed using the examination procedures. Two factors may account for this inconsistency; a) the reference standard for diagnosis (epidural blockade) is typically used as a therapy but is less favoured as a diagnostic tool [8,65]. In the context of this study the type of epidural injection (caudal, translaminar or transforaminal) and response criteria were not standardized. b) The criteria for clinical diagnosis were rather loose and consisted of dominant pain in the lower extremity aggravated by either the straight-leg-raise or femoral nerve tension tests. Further studies are required to improve the precision and clarity of the criteria for clinically classifying nerve root pain. Definitional distinctions between radiculopathy, radicular pain and somatic referred pain are clear, but it is unknown whether recommended clinical criteria can reliably distinguish between these concepts. This study did not attempt to explore these issues.

On the surface, agreement does appear to be weak even though better than chance. However, this does not concern us greatly. This is a pragmatic report of the overall performance of a low tech clinical examination and diagnoses achieved, compared to highly sophisticated and predominantly invasive procedures with a complex group of patients. The high proportion of cases deemed to be diagnostically indeterminate or displaying confounding illness behaviours (in the opinion of the clinicians) attests to the complexity. The 50% agreement on patho-anatomic categories achieved in this study was about what was hoped for prior to commencement of these studies. As a consequence of this work, certain significant modifications to the clinical examination can be made and subsequent studies may demonstrate an improvement on what we achieved here. 'Exact agreement' is a very demanding requirement when multiple pathologies are present. 'Clinical agreement' as described in the paper is really all that can be expected of a low tech clinical examination of patients with low back pain – a symptom commonly described as 'non-specific' in the low back pain literature.

## Conclusion

Using available reference standard technique, two thirds of patients received a pathoanatomic diagnosis with multiple pain generators identified in 10% of cases. Diagnoses of the tissue origin of chronic LBP or referred lower extremity symptoms by experienced physiotherapy clinicians agreed with available reference standard diagnoses 19–24% over and above expected chance agreement.

## Competing interests

The authors declare that they have no competing interests. All authors declare that their contributions to this paper have been independent of influence from funding agencies

## Authors' contributions

Concept and research design provided by ML, CA, BÖ and HT. Project management provided by ML. Facilities and equipment provided by CA. Writing provided by ML, BÖ and BM with manuscript review by HT. Data analysis, statistical support and manuscript review provided by BM. All authors read and approved the final manuscript.

## Pre-publication history

The pre-publication history for this paper can be accessed here:



## Supplementary Material

Additional File 1 Flow diagram of patient recruitment patterns in the project. Click here for file

Additional File 2 Table 1. Demographic, medical and psychometric profile of chronic low back pain patients. Click here for file

Additional File 3 Table 2. Cross tabulation for physiotherapist and reference standard / expert opinion diagnoses Click here for file

Additional File 4 Cross-tabulation of reference standard / expert opinion and physiotherapy diagnostic groups Click here for file

Additional File 5 Appendix 1. Description of reference standards used in pathoanatomic diagnoses in low back pain Click here for file
