# Supplementary material for: Agreement between diagnoses reached by clinical examination and available reference standards: a prospective study of 216 patients with lumbopelvic pain
Source: BMC Musculoskelet Disord. 2005 Jun 9;6:28. doi: 10.1186/1471-2474-6-28 (PMC1184083; doi:10.1186/1471-2474-6-28)
Supplement: Additional File 1 — Flow diagram of patient recruitment patterns in the project. [file 1471-2474-6-28-S1.doc]

## Table 1. Demographic, medical and psychometric profile of chronic low back pain patients.

| **Variable** | No. | Mean | STD | SE Mean | Range | IQR |
| --- | --- | --- | --- | --- | --- | --- |
| Age | 216 | 44.2 | 13.1 | 0.89 | 20,77 | 17.0 |
| Duration (weeks) | 215 | 157.9 | 229.3 | 15.6 | 1,2184 | 134.0 |
| Time off work (weeks) | 101 | 114 | 138.5 | 13.8 | 1,884 | 93.0 |
| 100mm VAS (current pain intensity) | 216 | 57.7 | 24.7 | 1.68 | 0,108 | 37.8 |
| 100mm VAS (pain intensity at best) | 214 | 32.8 | 23.2 | 1.58 | 0,98 | 35.0 |
| 100mm VAS (pain intensity at worst) | 216 | 87.6 | 12.6 | 0.86 | 28,113 | 13.0 |
| Roland Morris 23 point Questionnaire [66] | 215 | 18.5 | 4.5 | 0.31 | 3,23 | 6.0 |
| Zung Depression Index Questionnaire [67] | 215 | 30.6 | 11.9 | 0.81 | 0,62 | 16.0 |
| MSPQ Questionnaire [68] | 214 | 10.0 | 7.0 | 0.48 | 0,29 | 11.0 |
|  |  | % |  | | | |
| Male | 123 | 56.9 |
| Smoker | 85 | 39.5 |
| Off work | 110 | 51.2 |
| Previous spinal surgery | 62 | 28.8 |
| Traumatic onset | 157 | 73.0 |
| Disabled (Roland–Morris ≥19) [69] | 128 | 59.5 |
| Not distressed –DRAM [70] “normal” | 26 | 12.0 |
| Not distressed –DRAM “at risk” | 80 | 37.0 |
| Distressed –DRAM “depressed” | 95 | 44.0 |
| Distressed –DRAM “somatic” | 14 | 6.5 |

Notes:

1. VAS = Visual Analog Scale
2. MSPQ = Modified Somatic Perception Questionnaire
3. DRAM = Distress Risk Assessment Method
4. No.=Number
5. STD = standard deviation
6. SE mean =- standard error of the mean
7. IQR = interquartile range
