# Supplementary material for: Agreement between diagnoses reached by clinical examination and available reference standards: a prospective study of 216 patients with lumbopelvic pain
Source: BMC Musculoskelet Disord. 2005 Jun 9;6:28. doi: 10.1186/1471-2474-6-28 (PMC1184083; doi:10.1186/1471-2474-6-28)
Supplement: Additional File 2 — Table 1. Demographic, medical and psychometric profile of chronic low back pain patients. [file 1471-2474-6-28-S2.doc]

## Table 2. Cross tabulation for physiotherapist and reference standard / expert opinion diagnoses

|  | | Reference standard diagnostic categories | | | | | | | | | | | | | | | | | | | | | | | | | | |  |
| --- | --- | --- | --- | --- | --- | --- | --- | --- | --- | --- | --- | --- | --- | --- | --- | --- | --- | --- | --- | --- | --- | --- | --- | --- | --- | --- | --- | --- | --- |
|  |  | D | DF | DH | DJ | DL | DR | DRL | DRS | DT | F | FH | FL | FR | FS | H | J | JL | L | LN | N | O | R | RN | RO | RS | S | T | TOT |
| Physiotherapy diagnostic categories | D | 33 | 0 | 0 | 1 | 5 | 3 | 0 | 1 | 0 | 4 | 0 | 0 | 0 | 0 | 0 | 1 | 1 | 3 | 5 | 13 | 0 | 5 | 1 | 1 | 0 | 2 | 0 | 79 |
|  | DH | 0 | 0 | 1 | 0 | 0 | 0 | 0 | 0 | 0 | 0 | 0 | 0 | 0 | 0 | 0 | 0 | 0 | 0 | 0 | 0 | 0 | 0 | 0 | 0 | 0 | 0 | 0 | 1 |
|  | DL | 2 | 0 | 0 | 0 | 0 | 1 | 1 | 0 | 0 | 0 | 0 | 0 | 0 | 0 | 0 | 0 | 0 | 0 | 1 | 0 | 0 | 0 | 0 | 0 | 0 | 0 | 0 | 5 |
|  | DR | 2 | 0 | 0 | 0 | 0 | 1 | 0 | 0 | 0 | 0 | 0 | 0 | 0 | 0 | 0 | 0 | 0 | 0 | 0 | 2 | 0 | 3 | 0 | 0 | 1 | 0 | 0 | 9 |
|  | DT | 0 | 0 | 0 | 0 | 0 | 0 | 0 | 0 | 1 | 0 | 0 | 0 | 0 | 0 | 0 | 0 | 0 | 0 | 0 | 0 | 0 | 0 | 0 | 0 | 0 | 0 | 0 | 1 |
|  | F | 2 | 0 | 0 | 0 | 0 | 0 | 0 | 0 | 0 | 4 | 0 | 0 | 0 | 0 | 0 | 0 | 0 | 0 | 1 | 0 | 1 | 1 | 0 | 0 | 0 | 0 | 0 | 9 |
|  | FL | 0 | 0 | 0 | 0 | 0 | 0 | 0 | 0 | 0 | 0 | 0 | 0 | 0 | 0 | 0 | 0 | 0 | 0 | 1 | 0 | 0 | 0 | 0 | 0 | 0 | 0 | 0 | 1 |
|  | H | 0 | 0 | 0 | 0 | 0 | 0 | 0 | 0 | 0 | 0 | 1 | 0 | 0 | 0 | 3 | 0 | 0 | 0 | 0 | 0 | 0 | 0 | 0 | 0 | 0 | 0 | 0 | 4 |
|  | HO | 0 | 0 | 0 | 0 | 0 | 0 | 0 | 0 | 0 | 0 | 0 | 0 | 0 | 0 | 0 | 0 | 0 | 0 | 0 | 0 | 1 | 0 | 0 | 0 | 0 | 0 | 0 | 1 |
|  | J | 1 | 0 | 0 | 0 | 0 | 0 | 0 | 0 | 0 | 0 | 0 | 0 | 0 | 0 | 0 | 2 | 0 | 0 | 0 | 1 | 0 | 0 | 0 | 0 | 0 | 1 | 0 | 5 |
|  | JT | 0 | 0 | 0 | 0 | 0 | 0 | 0 | 0 | 0 | 0 | 0 | 0 | 0 | 0 | 0 | 0 | 1 | 0 | 1 | 0 | 0 | 0 | 0 | 0 | 0 | 0 | 0 | 2 |
|  | L | 0 | 0 | 0 | 0 | 2 | 0 | 0 | 0 | 0 | 0 | 0 | 0 | 0 | 0 | 0 | 0 | 0 | 1 | 6 | 4 | 0 | 0 | 0 | 0 | 0 | 1 | 0 | 14 |
|  | LN | 6 | 2 | 0 | 0 | 0 | 0 | 0 | 0 | 0 | 0 | 0 | 0 | 0 | 0 | 1 | 0 | 0 | 3 | 14 | 2 | 1 | 1 | 0 | 0 | 0 | 1 | 0 | 31 |
|  | N | 7 | 0 | 0 | 0 | 2 | 1 | 0 | 0 | 0 | 5 | 0 | 1 | 0 | 0 | 1 | 0 | 0 | 3 | 5 | 5 | 0 | 1 | 0 | 0 | 0 | 1 | 0 | 32 |
|  | O | 0 | 0 | 0 | 0 | 0 | 0 | 0 | 0 | 0 | 0 | 0 | 0 | 0 | 0 | 0 | 0 | 0 | 0 | 0 | 1 | 0 | 0 | 0 | 0 | 1 | 0 | 0 | 2 |
|  | R | 4 | 0 | 0 | 0 | 1 | 3 | 0 | 0 | 0 | 0 | 0 | 0 | 0 | 1 | 0 | 0 | 0 | 0 | 0 | 0 | 0 | 2 | 0 | 0 | 0 | 0 | 0 | 11 |
|  | RT | 1 | 0 | 0 | 0 | 0 | 0 | 0 | 0 | 0 | 0 | 0 | 0 | 0 | 0 | 0 | 0 | 0 | 0 | 0 | 0 | 0 | 0 | 0 | 0 | 0 | 0 | 0 | 1 |
|  | S | 1 | 0 | 0 | 0 | 0 | 0 | 0 | 0 | 0 | 1 | 0 | 0 | 2 | 1 | 0 | 0 | 0 | 0 | 0 | 0 | 0 | 0 | 0 | 0 | 0 | 2 | 0 | 7 |
|  | SN | 0 | 0 | 0 | 0 | 0 | 0 | 0 | 0 | 0 | 0 | 0 | 0 | 0 | 0 | 0 | 0 | 0 | 0 | 0 | 0 | 0 | 0 | 0 | 0 | 0 | 0 | 1 | 1 |
|  | TOT | 59 | 2 | 1 | 1 | 10 | 9 | 1 | 1 | 1 | 14 | 1 | 1 | 2 | 2 | 5 | 3 | 2 | 10 | 34 | 28 | 3 | 13 | 1 | 1 | 2 | 8 | 1 | 216 |

Notes: D = disk, F=facet/Z joint, J=SIJ, R=nerve root, H=hip, S=stenosis, L=illness behaviour, N=indeterminate, O=other, T=instability, TOT=Totals
