# Supplementary material for: Agreement between diagnoses reached by clinical examination and available reference standards: a prospective study of 216 patients with lumbopelvic pain
Source: BMC Musculoskelet Disord. 2005 Jun 9;6:28. doi: 10.1186/1471-2474-6-28 (PMC1184083; doi:10.1186/1471-2474-6-28)
Supplement: Additional File 3 — Table 2. Cross tabulation for physiotherapist and reference standard / expert opinion diagnoses [file 1471-2474-6-28-S3.doc]

**Table 3. Cross-tabulation of reference standard / expert opinion and physiotherapy diagnostic groups**

|  | | **Reference standard / expert opinion diagnostic groups** | | | | | | | | | |
| --- | --- | --- | --- | --- | --- | --- | --- | --- | --- | --- | --- |
|  |  | Disk | ZJ | SIJ | N.Root | Hip | Stenosis | Other | Illness B | Indeterm | Totals |
| **Physiotherapy diagnostic groups** | Disk | **52** | 4 | 3 | 18 | 1 | 4 | 2 | 16 | 22 | 122 |
|  | ZJ | 2 | **4** | 0 | 1 | 0 | 0 | 1 | 2 | 2 | 12 |
|  | SIJ | 1 | 0 | **2** | 0 | 0 | 1 | 0 | 1 | 2 | 7 |
|  | N.Root | 13 | 1 | 0 | **11** | 0 | 2 | 1 | 2 | 2 | 32 |
|  | Hip | 1 | 1 | 0 | 0 | **5** | 0 | 1 | 0 | 0 | 8 |
|  | Stenosis | 1 | 4 | 0 | 2 | 0 | **3** | 1 | 0 | 0 | 11 |
|  | Other | 2 | 0 | 0 | 1 | 0 | 1 | **2** | 1 | 2 | 9 |
|  | Illness B | 13 | 2 | 0 | 2 | 1 | 2 | 0 | **28** | 28 | 76 |
|  | Indeterm | 18 | 8 | 1 | 3 | 2 | 2 | 2 | 29 | **26** | 91 |
|  | Totals | 103 | 24 | 6 | 38 | 9 | 15 | 10 | 79 | 84 | 368 |

Notes:

1. Bolded figures represent counts for agreement
2. Disk = discogenic pain, ZJ = zygapophysial (facet joint pain), SIJ = sacroiliac joint pain, N.Root, nerve root (radicular) pain, Hip = hip joint pain, Stenosis = spinal stenosis, Other = Other diagnoses, Illness B = Illness behaviours, Indeterm = Diagnostically Indeterminate.
