# Supplementary material for: Agreement between diagnoses reached by clinical examination and available reference standards: a prospective study of 216 patients with lumbopelvic pain
Source: BMC Musculoskelet Disord. 2005 Jun 9;6:28. doi: 10.1186/1471-2474-6-28 (PMC1184083; doi:10.1186/1471-2474-6-28)
Supplement: Additional File 4 — Cross-tabulation of reference standard / expert opinion and physiotherapy diagnostic groups [file 1471-2474-6-28-S4.doc]

## Appendix 1. Reference standards used in pathoanatomic diagnoses in low back pain

|  | **Diagnosis** | **Reference standard** |
| --- | --- | --- |
| **Patho-anatomic diagnoses** | Discogenic pain | Provocation discography [45] |
|  | ZJ Pain | Intra-articular or medial branch block [11] |
|  | SIJ pain | Intra-articular block [13] |
|  | Nerve root / dural pain | Sciatica relieved by selective epidural injection with appropriate CT or MRI findings [74] |
|  | Claudication associated with spinal stenosis | Neurogenic claudication with CT or MRI imaging [29,80] |
|  | Hip joint pain | Intra-articular block [81,82] |
|  | Others | Imaging and other appropriate diagnostic methods (e.g. fractures, neoplasm) |
| **Non-anatomic** classifications | Illness behaviour | Questionnaires, clinician opinion [66] |
|  | Instability | “paradoxical motion” flexion/extension radiographs [35] |
|  | Indeterminate | Diagnosis impossible or diagnostic procedures confounded |

Notes: ZJ = zygapophysial joint, SIJ = sacroiliac joint, ^1^Stenosis refers to symptomatic spinal stenosis
