# Supplementary material for: Agreement between diagnoses reached by clinical examination and available reference standards: a prospective study of 216 patients with lumbopelvic pain
Source: BMC Musculoskelet Disord. 2005 Jun 9;6:28. doi: 10.1186/1471-2474-6-28 (PMC1184083; doi:10.1186/1471-2474-6-28)
Supplement: Additional File 5 — Appendix 1. Description of reference standards used in pathoanatomic diagnoses in low back pain [file 1471-2474-6-28-S5.doc]

**Appendix 2. Summary of diagnostic accuracy of key clinical tests used in the study available at the commencement of the study.**

| **Disorder** | **Test / Sign / Variable** | **%Sensitivity** | **%Specificity** | **References** |
| --- | --- | --- | --- | --- |
| Lumbar HNP | Ipsilateral SLR | 76-97 | 11-45 | [71] [72] [73] |
|  | contralateral SLR | 23-27 | 88-100 | [71] [72] [74] [73] |
|  | Weakness ankle dorsiflexion | 20-49 | 54-82 | [71] [72] |
|  | Weakness EHL | 0.37 | 0.71 | [72] |
|  | Ankle reflex weak | 50-52 | 62-63 | [71] [72] |
|  | Sensory loss | 0.66 | 0.51 | [73] |
|  | Patellar reflex weak | 40-70 | 93-97 | [71] [72] |
|  | Quads weakness | 0.10 | 0.99 | [72] |
|  | Ankle PF weakness | 0.60 | 0.95 | [72] |
| LSP disc / positive discography | Centralization or peripheralisation | 0.94 | 0.52 | [22,75] |
|  | Centralization | 0.92 | 0.64 | [22](calculations based on data provided) |
|  | Peripheralization | 0.69 | 0.64 | [22](calculations based on data provided) |
| Lumbar facet | Extension / rotation | 1.00 | 0.12 | [76] |
| (single blocks) | 5/7 Revel criteria | 1.00 | 0.66 | [25] |
| (single blocks) | 5/7 including good relief lying | 0.92 | 0.80 | [25] |
| (double blocks) | 5/7 Revel criteria | 0.13 | 0.84 | [57] |
| (double blocks) | Extension/rotation | 0.31 | 0.69 | [57] |
| Symptomatic | Age > 65 | 0.77 | 0.69 | [77] |
| Spinal stenosis | No pain when seated | 0.46 | 0.93 | [77] |
|  | Sx improved when seated | 0.52 | 0.83 | [77] |
|  | Sx worse when walking | 0.71 | 0.30 | [77] |
|  | Better walking with shop cart | 0.63 | 0.67 | [78] |
|  | leg pain worse walking better sitting | 0.81 | 0.16 | [78] |
|  | best sitting | 0.89 | 0.39 | [78] |
|  | worst standing/walking | 0.89 | 0.33 | [78] |
|  | no pain lumbar flexion | 0.79 | 0.44 | [77] |
|  | thigh pain with 30secs extension | 0.51 | 0.69 | [77] |
|  | increase walking distance with spinal flexion | 0.58 | 0.91 | [79] |
|  | increase walking distance on inclined treadmill | 0.68 | 0.83 | [78] |
